# Chronic Fatigue in Cancer, Brain Connectivity and Reluctance to Engage in Physical Activity: A Mini-Review

**DOI:** 10.3389/fonc.2021.774347

**Published:** 2021-12-20

**Authors:** Nathalie André, Steven Gastinger, Amélie Rébillard

**Affiliations:** ^1^ Research Centre on Cognition and Learning (UMR CNRS 7295), University of Poitiers, Sport Sciences Faculty, Poitiers, France; ^2^ Maison des Sciences de l’Homme et de la Société (USR CNRS 3565), Université de Poitiers, Poitiers, France; ^3^ M2S-EA7470, University of Rennes, Rennes, France; ^4^ APCoSS - Institut de Formation en Education Physique et en Sport (IFEPSA), UCO Angers, Angers, France; ^5^ Institut Universitaire de France (IUF), Paris, France

**Keywords:** chronic fatigue, immediate benefits, functional connectivity, physical activity, effort-based decision making, cost-benefit analysis

## Abstract

A large amount of evidence shows that after a cancer diagnosis, patients significantly reduce their level of physical activity. Usually, this reduction is attributed to cancer-related fatigue. However, to our knowledge, no study has clearly demonstrated that fatigue alters effort-based decision-making in cancer. This mini-review aimed to provide evidence that chronic fatigue in cancer patients causes changes in brain connectivity that impact effort-based decision-making. Indeed, three patterns of activation to compensate for dysfunctional networks have been reported: greater variability in the executive network and hyperactivation in the executive network, which account for less efficient and costly processes in the frontal cortex, and reduced deactivation in the default mode network. Nevertheless, these activation patterns are also observed with other factors, such as anticipatory stressors (worry, rumination or sleep loss), that might also cause reluctance to engage in physical activity. Effort-based decision-making involving weighing costs against benefits and physical activity interventions should increase immediate benefits to facilitate engagement in effortful activities.

## Introduction

Cancer-related fatigue (CRF) is one of the most common and distressing side effects and can persist for years after treatment has ended in otherwise healthy survivors (1). This feeling of fatigue is generally perceived as the main impediment to physical activity (PA), which requires physical and mental effort. Indeed, walking regularly, muscle-strengthening activities, maintaining intensity throughout a physical activity program and planning exercise for the following weeks are mentally and physically costly. Moreover, a general belief among cancer patients, family caregivers and some health professionals is that fatigue is recovered by rest rather than activity (2). Therefore, unsurprisingly, the literature on PA and CRF frequently reports that the level of PA after a cancer diagnosis or during cancer treatment is significantly reduced, all cancer combined [e.g (3–5)].

However, the special hallmark of fatigue in cancer is that it is not recovered by rest but rather by activity. Indeed, an increasing number of meta-analyses and systematic reviews report that PA is more effective than rest for reducing fatigue, suggesting a moderate effect [e.g (6, 7)]. Specifically, beneficial effects of exercise on fatigue have been observed in trials conducted with patients during and after treatment (8–10), indicating that resistance and moderate-to-high-intensity exercises can be helpful at different stages of the disease trajectory. During treatment, exercise may buffer treatment-related increases in fatigue, whereas exercise may reduce fatigue in patients after treatment completion [e.g (6, 11)]. In other words, PA allows maintenance of a baseline level of fatigue (generally measured after diagnosis) and faster recovery from treatment (8–10, 12–17). However, exercise programs require effortful engagement, which may explain the low level of adherence and the high level of attrition observed in clinical trials (18–21).

If cancer patients are reluctant to engage in PA despite being aware of the long-term benefits of PA, something may be missing in our understanding of CRF and its role in effort-based decision-making regarding participation in PA (22–24). The decision to engage in PA relies on the computation of several costs of effort and expected benefits of PA (25, 26), and chronic fatigue might be a key aspect of unwillingness to expend any effort (24, 27, 28). Chronic fatigue can hinder motivation or self-regulatory capacities, leading cancer patients to choose effortless activities such as rest rather than effortful activities such as PA.

**Figure 1:**
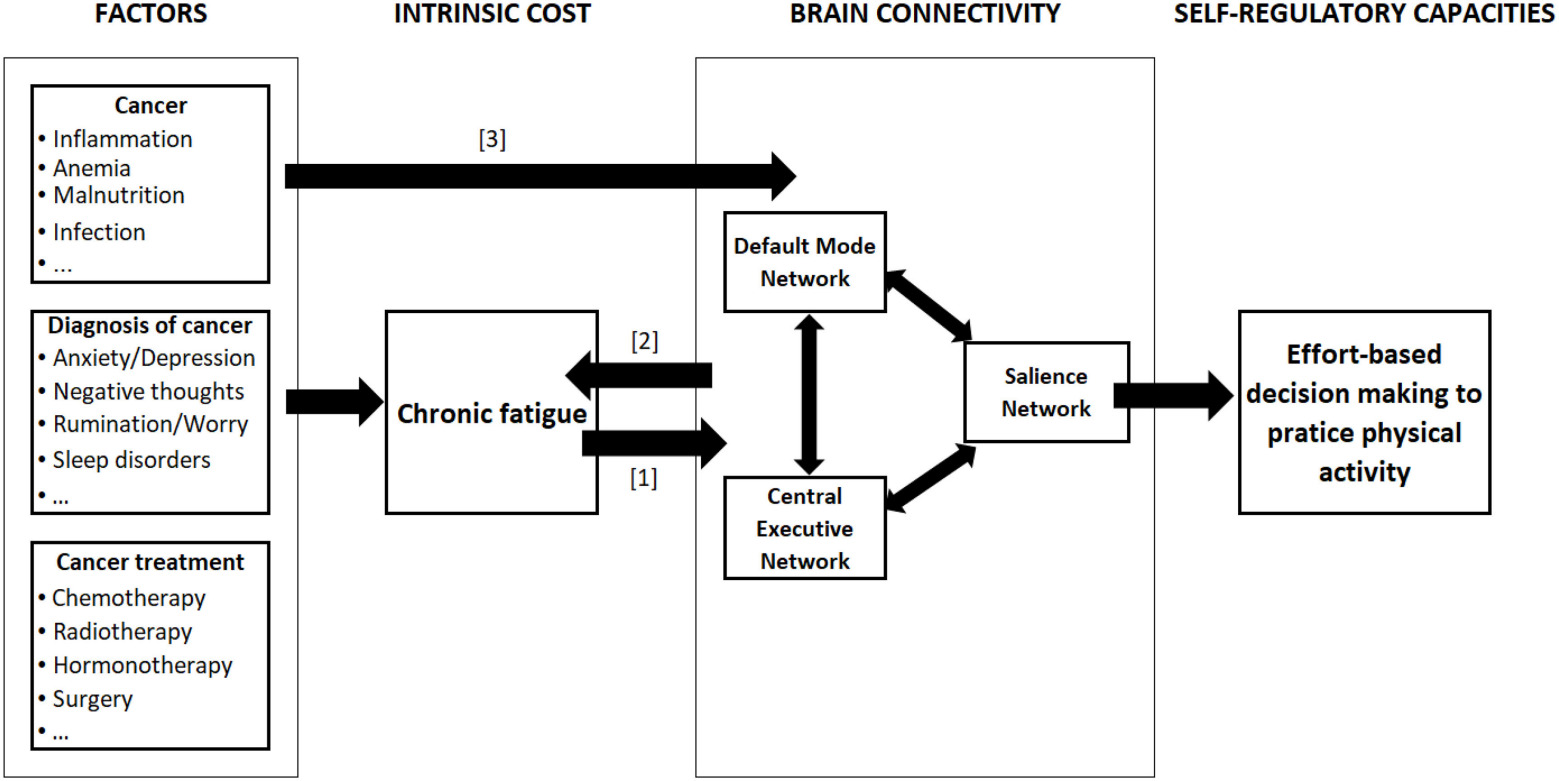
Relationships between chronic fatigue and brain connectivity. The figure represents the links between cancer-related factors and effort-based decision-making to practice PA. The causal relationship between chronic fatigue and brain connectivity (BC) can be of three types: [1] fatigue causes changes in BC; [2] the change in BC causes fatigue; [1&2] a bidirectional relationship. Moreover, factors such as cancer itself, diagnosis and treatment of cancer may directly alter the brain connectivity [3].

An interesting opportunity to achieve substantial progress in PA-based interventions for fatigued cancer patients would be to examine brain connectivity and its effect on effort-based decision-making. Indeed, to date, concerns have focused on the role of inflammation in the development of CRF (24, 29–32), but its implication in the reluctance of cancer patients to engage in PA has not been established. Even if several factors can cause durable changes in brain functional connectivity (See **Figure 1**), we propose that brain connectivity dysfunction in case of chronic fatigue alters effort-based decision-making useful to engage in PA. This hypothesis has never been proposed.

Here, we demonstrate that CRF is associated with alterations in brain connectivity related to effort-based decision-making and can thus hinder attempts to engage in regular PA. Accordingly, we demonstrate that fatigue in cancer alters such brain connectivity by examining studies using functional magnetic resonance imaging (fMRI). The discussion section highlights that interventions targeting chronic fatigue in cancer should consider individual differences in lost or depleted inner resources rather than symptoms alone, as in previous cancer research. Clinical and interventional implications are then proposed.

## Definition of Chronic Fatigue in Cancer

Although no definition of CRF has been established, the concept has been described. According to the National Comprehensive Cancer Network (33), CRF is described as “a distressing, persistent, subjective sense of physical, emotional, and/or cognitive tiredness or exhaustion related to cancer or cancer treatment that is not proportional to recent activity and interferes with functioning”. Cancer-related fatigue should be distinguished from everyday fatigue, which can be easily recovered by rest. According to Bootsma et al. (34), CRF is chronic and should be considered a permanent symptom. Nevertheless, CRF is increasingly regarded as a multisymptom concept generally associated with physical and mental fatigue rather than a single symptom (35, 36). Thus, fatigue in cancer refers to a failure to initiate and/or sustain attentional tasks (mental fatigue) and physical activities (physical fatigue) requiring self-motivation (as opposed to external stimulation) in the absence of any clinically detectable motor weakness or dementia (37).

## Brain Network and Functional Connectivity in Chronic Fatigue

### Three Large-Scale Brain Networks

Deciding to practice regular PA according to recommendations requires efficient cognitive control and self-regulatory capacities. Neuroimaging studies analyzing resting-state functional connectivity have suggested the existence of at least three large-scale brain networks related to different aspects of high-level cognitive functions and self-regulation (38–41), including the default-mode network (DMN), the central executive network (CEN) and the salience network (SN).

The DMN plays a key role in self-related processes, introspection, self-awareness, metacognition, prospective self-projection, and autobiographic memory recall (42–47) and is deactivated during cognitively demanding tasks (48–51). The DMN thus allows the construction of mental models or simulations having an adaptive function and facilitating future behavior. These simulations therefore represent a means of anticipating and evaluating future events to react to them as best as possible, to build a stable identity over time and to adapt to the social world. The DMN mainly includes the ventromedial prefrontal cortex, the posterior cingulate cortex, the precuneus, the retrosplenial cortex, the lateral parietal lobes and the medial temporal lobes (52).

The CEN and SN are activated during a wide variety of tasks (40, 49). The CEN (36, 53), a frontoparietal cognitive system that controls and manages executive functions, contributes to executive control, particularly by maintaining and updating information in working memory, sustained attention, response selection, and response suppression. The CEN is also responsible for decision-making and problem solving in the pursuit of goal-directed behavior (41). The CEN is mainly anchored in the dorsolateral prefrontal cortex, the ventrolateral prefrontal cortex, the dorsomedial prefrontal cortex, and the lateral posterior parietal cortex (40, 53, 54).

The SN, a cingulated frontal operculum system, is involved in identifying the most homeostatically relevant signals among a myriad of internal and extrapersonal stimuli to make decisions (40, 55), manage errors and conflicts (56, 57), and ensure autonomic control (58, 59). The salience network is a large functional neuronal network involved in the generation of the effort signal (22). This system mainly consists of the orbital frontoinsular cortex, the dorsal anterior cingulated cortex (dACC), the anterior insula, and the superior temporal gyrus (40). Uddin et al. (60) reported that during task performance, the salience network coordinates and controls deactivation of the DMN and activation of the CEN.

### Functional Connectivity in Chronic Fatigue

Several studies using brain imaging in humans confirm that the within-network connectivity of the SN is weakened in the case of chronic fatigue syndrome/myalgic encephalomyelitis and depression (61–66). In addition, these disorders are associated with persistent weakened connectivity between the SN and CEN and persistent strengthened connectivity between the SN and DMN (66–68). This deterioration in effort capacity results in a persistent weakening of SN connectivity through negative long-term structural changes requiring several weeks or months to occur and a brain connectivity less coordinated across area of the DMN (69, 70). The following section provides evidence that altered brain connectivity in chronic fatigue in cancer hinders effort-based decision-making.

## Task-Related Functional Connectivity and Chronic Fatigue in Cancer

The question is whether chronic fatigue in cancer alters functional brain connectivity implied in effort-based decision-making; if so, a new mechanism related to brain connectivity explaining disengagement from PA by cancer patients warrants investigation. Based on six studies examining the relationship between chronic fatigue and task-related fMRI (71–76), three patterns of connectivity have been identified as plausible neural biomarkers of chronic fatigue in cancer patients (See **Table 1**).

**Table 1:** Characteristics of included studies.

References	Population	• Design • Type of cancer • Moment of the treatment	Measure of fatigue	fMRI scanner task	Patterns of brain functional connectivity	Relationship with chronic fatigue
**Churchill et al. (****76****)**	28 women in pre-CHE 37 women pre-RAD 32 healthy control N/A	**• **Cross-sectional **• **Breast cancer (24 to 34 days after surgery) **• **Pre-treatment	The Functional Assessment of Chronic Illness Therapy - Fatigue (FACIT-F)	Verbal working memory task	↑ of the activation in posterior cingulate cortex and precuneus activation and ↓ in Hurst exponent (parietal lobes and thalamus) in high distress profile (patients with sleep problems and worry).	No direct measure of fatigue but the physical distress (fatigue and sleep disorders) is correlated with the decrease in Hurst exponent.
**Askren et al. (****71****)**	28 women with CHE 37 women non CHE 32 healthy control 51 years old	**• **Cross-sectional **• **Breast cancer **• **During treatment	The Functional Assessment of Chronic Illness Therapy - Fatigue (FACIT-F)	Verbal working memory task	↑ spatial variance in executive network (left and right frontal cortex, anterior cingulate, left and right parietal cortex) for the CHE groups compared to the two other groups.	The spatial variance in executive network predicts post-treatment fatigue severity and cognitive complaints for CHE group
**Menning et al. (****74****)**	32 women in pre-CHE 33 women pre-non CHE 38 healthy control 50.9 years old	**• **Cross-sectional **• **Breast cancer **• **Pre-treatment	Fatigue subscale of the EORTC QLQ-C30 Fatigue subscale of the POMS	**• **Planning test (Tower of London) **• **Paired Associates memory task	Pre-CHE vs Control groups: hyperactivation of the dorsomedial prefrontal cortex extending into the DLPFC with increasing task difficulty for pre-CHE. Pre-non CHE vs Control groups: subthreshold hyperactivation of the dorsomedial prefrontal cortex extending into the DLPFC for pre-non CHE.	Significant correlation between fatigue and the activation of the dorsomedial prefrontal cortex on the planning task across all groups.
**Menning et al. (****75****)**	28 women with CHE (with or without endocrine treatment) 24 women non CHE 31 healthy control 50.6 years old	**• **Cross-sectional **• **Breast cancer **• **During treatment	Fatigue subscale of the EORTC QLQ-C30	**• **Planning test (Tower of London) **• **Paired Associates memory task	CHE vs non CHE: ↑ of the activation in the bilateral inferior parietal cortex and the precuneus extending into the superior parietal cortex with increasing task load in the CHE compared to the non CHE group. Non CHE vs Control: ↓ over time of the activation in the right inferior parietal cortex in non CHE compared to Control group with increasing task load.	The non CHE group showed a correlation between baseline fatigue and change in BOLD signal in the right inferior parietal cortex during the planning task. A negative correlation was found between change in fatigue and change in BOLD signal in the right inferior parietal cortex in the non CHE group.
**Jung et al. (****72****)**	28 women with CHE 34 women non CHE 30 control group 51.58 years old	**• **Longitudinal **• **Breast cancer **• **4 moments: 1 month post-surgery; before any planned adjuvant; five months following baseline; 1 year post-baseline	Breast Cancer Prevention Trial Symptom Scales (BCPTSS)	Verbal working memory task	From baseline to Month 12: ↑ spatial variance in the executive network during task activation for the CHE group compared to the other groups. In healthy patient, the variance of the fMRI signal decrease during task activation (habituation).	No direct measures of fatigue but persistent cognitive complaints are correlated with physical symptom severity and worry regardless of treatment.
**De Dreu et al. (****73****)**	23 participants with meningioma 21 low grade glioma 19 high grade glioma 49.2 years old	**• **Cross-sectional **• **Brain tumor **• **After surgery	Multidimensional fatigue inventory (MFI-20)	Task on attention (analysis during phasic alertness)	↓ of the deactivation of the DMN (left and right lingual cortex; left and right cuneus; right precuneus) for the three groups.	The total score of fatigue do not correlate with the signal change in the CEN during phasic alertness while total score of fatigue, but also general, physical and mental fatigue correlate with the signal change in the DMN for each group.

CHE, chemotherapy; RAD, radiotherapy; EORTC-QLQ-C30, European Organization for Research and Treatment of Cancer-Quality of Life Questionnaire-C30; POMS, Profile Of Mood State.

### Spatial Variance in the Executive Network

First, using verbal working memory task-related fMRI, Askren et al. (71) and Jung et al. (72) compared women with breast cancer treated with chemotherapy to women who received no adjuvant treatment. They both reported increased variance in the BOLD signal in the executive network in women with adjuvant treatment. Typically, the neural response in healthy people is lower spatial variance in executive network activity during task activation, reflecting strong attentional engagement. Interestingly, this greater spatial variance was still higher in the posttreatment chemotherapy group than in the nontreatment group in both studies. In contrast, this change in variance predicted posttreatment fatigue only in the study by Askren et al. (71). Engagement of the executive (or frontoparietal) network in the case of chemotherapy was found to be more variable/idiosyncratic (77) in chemotherapy-treated women. Generally, this pattern of spatial activation is often considered a compensatory mechanism such that when a given neural system is dysfunctional, other systems (regions/networks) may become engaged in an attempt to support task performance. However, this variability consumes more biological resources and is costly.

### Hyperactivation of the Executive Network

In addition to the variability in the executive network, Menning et al. (74, 75) and De Dreu et al. (73) reported hyperactivation in the CEN using task-related fMRI (verbal working memory and planning tasks, respectively). This hyperactivation was mainly observed before and during treatment but not posttreatment. Pretreatment hyperactivity was higher in women awaiting chemotherapy than in those without chemotherapy. This pattern of connectivity was explained by fatigue only in the studies by Menning et al. (74, 75). In these three studies, chemotherapy-treated women had increased brain activation in the dorsolateral prefrontal cortex during the tasks, suggesting that the hub of the executive network may be overengaged during task processing. The hyperactivation in the CEN can be explained by increased recruitment of expanded neural circuitry to support structural functioning (78). Here, again, this pattern may be associated with a compensatory mechanism. Interestingly, CEN hyperactivation decreased over time in the chemotherapy group (i.e., in the posttreatment group), with increasing task loads corresponding to more difficulties sustaining mental effort.

These results suggest that during a task targeting executive functions, women with breast cancer must expend more effort due to greater inner resource depletion. This widespread activation may lead to an increased demand for neural resources such as oxygen and glucose, in turn leading to fatigue (79). Fatigue and lower performance have been associated with increased brain activity while performing a high-effort cognitive task (80–82). Severe fatigue has been hypothesized to consume a significant amount of attentional resources in terms of recruiting additional brain regions for cognitive compensation to perform better in tasks depending on the degree of mental effort (82).

### Reduced Deactivation of the DMN

A third pattern of connectivity emerged in three studies (73, 75, 76) on brain tumors and breast cancer during treatment and before chemotherapy. They all reported a reduced capacity to inhibit DMN activation, which was correlated with measures of fatigue. Given the role of the DMN in disrupting attentional and engagement processes (38), failure to suppress DMN regions during tasks should be related to decreased attention and/or motivation toward the task. Nevertheless, within the DMN, hyperactivation was also observed on resting-state fMRI (62). Chemotherapy can induce white matter disruption (83) and a reduction in gray matter density in several brain regions, including the DMN (84). Specifically, the precuneus, cingulate, lateral parietal cortex, medial frontal gyrus, cerebellum and hippocampus appear to be the structures most impacted by cancer treatments. Since the DMN is thought to be involved in contemplation, remembering, and rumination, the authors suggested that enhanced connectivity between the DMN and the frontal gyrus may be related to more cogitation (62) and partially responsible for mental fatigue.

This failure to suppress default mode activity during tasks has been linked to decreased activity in task-related regions leading to attentional lapses and decreases in performance (85–87). This disengagement of brain regions associated with mental effort (higher spatial variance) favoring of brain regions linked to resting activity (the default network) might be intended to conserve mental resources for the maintenance of engagement in the task (88).

These results show that the hypothesized pattern of connectivity is partly corroborated, that is, a reduced capacity of the SN to deactivate the DMN. In contrast, no clear weakening of the connectivity between the SN and the CEN has been reported. Nevertheless, the increasing activation of the CEN may be associated with a compensatory mechanism. Finally, reduced connectivity in the executive network is associated with fatigue and task performance failure after chemotherapy completion.

## Discussion

This mini-review aimed to explore the relationship between chronic fatigue in cancer and functional connectivity patterns during task-related fMRI. Our literature review suggests that effort-based decision-making may be altered by the changes in brain connectivity, specifically within DMN and between DMN and other networks. These results do not provide information on the direction of the relationships between chronic fatigue and network connectivity. Nevertheless, the direction of this relationship between these two variables can be conceived in three ways: a unilateral effect of chronic fatigue on network connectivity, a unilateral effect of changes in connectivity that induce chronic fatigue, or a bidirectional relationship between these two variables.

Chronic fatigue might result in difficulty concentrating (i.e., a symptom), which is explained by different brain activation patterns. First, the pattern of chronic fatigue without treatment or before treatment resembles alterations caused by anticipatory stress. Andreotti et al. (89) emphasized neurocognitive alterations in cancer populations independent of treatments, supporting the role of the allostatic load induced by recurrent and continuous stressors such as cancer diagnoses, negative recurrent thoughts, and chronic pain but also by remembering novel medication regimens and medical appointments. The allostatic system includes two established large-scale brain networks containing most of the limbic cortices: the SN and the DMN. According to the authors, the allostatic mechanisms are not tuned to help an individual respond effectively to prolonged and repetitive psychological stressors that lead to overactivity and dysregulation of the allostatic network. Interestingly, Brosschot et al. (90) proposed that this prolonged activation can also be generated by prolonged active cognitive representations of stressors, also called perseverative cognition, and that it occurs by phenomena such as worry, rumination and anticipatory stress, such as a lack of sleep.

The dysfunctional activation of the executive network (i.e., greater spatial variance and hyperactivation of the executive network) validates the ability of cancer patients to use compensatory mechanisms to successfully engage in effortful activities but at the cost of significant effort. This capacity is reduced during treatment and at the end of the treatment, suggesting that the toxicity of the molecules during chemotherapy modifies the structure of the brain at the gray and white matter levels, which modifies functional brain connectivity. Thus, cancer patients are no longer be able to compensate.

In summary, chronic fatigue leads to the use of compensatory mechanisms that are cognitively costly for cancer patients, which might impact the decision to engage in or maintain PA. These mechanisms have been reported as patterns of adaptation of brain activity in studies examining the functional connectivity in several chronic diseases such as chronic fatigue syndrome (69) and multiple sclerosis (91). However, this hyperactivation is not always correlated with the level of fatigue. In contrast, the reduced deactivation of the DMN in the three studies examined appears to correlate with the level of fatigue. According to Shan et al. (69), deficits in DMN could be energy expensive and may contribute to or cause the fatigue. This abnormal activity of DMN might be a marker of fatigue in cancer.

Three perspectives can be proposed. First, PA interventions should increase immediate benefits to reduce the imbalance between cost (chronic fatigue) and benefits (general wellbeing) and facilitate engagement in effortful activities such as PA. Interestingly, behavioral change techniques have been reported to be helpful interventions to increase immediate benefits such as ‘social rewards’, ‘prompts’, ‘nonspecific rewards’ and ‘graded tasks’ in cancer (92, 93). Second, exploring other regions of interest, such as the basal ganglia, may be more productive to understand the role of motivation and reward in effort-based decision-making among fatigued cancer patients (94). Decreased activation in the basal ganglia in chronic fatigue syndrome has been demonstrated to be correlated with increased mental fatigue, general fatigue and reduced PA (95). Third, these patterns of impaired brain connectivity in the case of chronic fatigue can also be observed with perseverative cognition (89), including worry, rumination and anticipatory stress, such as sleep disturbance. These three perspectives should be examined further.

## Author Contributions

NA, SG, and AR conceived the plan of the article and were engaged in the literature review process. NA wrote the article. NA, SG, and AR reread the last version of the article. All authors contributed to the article and approved the submitted version.

## Conflict of Interest

The authors declare that the research was conducted in the absence of any commercial or financial relationships that could be construed as a potential conflict of interest.

## Publisher’s Note

All claims expressed in this article are solely those of the authors and do not necessarily represent those of their affiliated organizations, or those of the publisher, the editors and the reviewers. Any product that may be evaluated in this article, or claim that may be made by its manufacturer, is not guaranteed or endorsed by the publisher.
